# Data‐Driven Intelligent Manipulation of Particles in Microfluidics

**DOI:** 10.1002/advs.202205382

**Published:** 2022-12-20

**Authors:** Wen‐Zhen Fang, Tongzhao Xiong, On Shun Pak, Lailai Zhu

**Affiliations:** ^1^ Department of Mechanical Engineering National University of Singapore Singapore 117575 Singapore; ^2^ Key Laboratory of Thermo‐Fluid Science and Engineering MOE, Xi'an Jiaotong University Xi'an 710049 China; ^3^ Department of Mechanical Engineering Santa Clara University Santa Clara CA 95053 USA

**Keywords:** artificial neural network, control, hydrodynamic interaction, machine learning, microfluidics

## Abstract

Automated manipulation of small particles using external (e.g., magnetic, electric and acoustic) fields has been an emerging technique widely used in different areas. The manipulation typically necessitates a reduced‐order physical model characterizing the field‐driven motion of particles in a complex environment. Such models are available only for highly idealized settings but are absent for a general scenario of particle manipulation typically involving complex nonlinear processes, which has limited its application. In this work, the authors present a data‐driven architecture for controlling particles in microfluidics based on hydrodynamic manipulation. The architecture replaces the difficult‐to‐derive model by a generally trainable artificial neural network to describe the kinematics of particles, and subsequently identifies the optimal operations to manipulate particles. The authors successfully demonstrate a diverse set of particle manipulations in a numerically emulated microfluidic chamber, including targeted assembly of particles and subsequent navigation of the assembled cluster, simultaneous path planning for multiple particles, and steering one particle through obstacles. The approach achieves both spatial and temporal controllability of high precision for these settings. This achievement revolutionizes automated particle manipulation, showing the potential of data‐driven approaches and machine learning in improving microfluidic technologies for enhanced flexibility and intelligence.

## Graphical abstract

Automated particle manipulation in microfluidics necessitates a physical model to describe particle motion. Such models are not accessible in general settings. This work presents a data‐driven approach integrating machine learning with feedback control, allowing a microfluidic device to learn the physical laws of particle kinematics in a general configuration. It is used to demonstrate hydrodynamic particle manipulations of high precision.

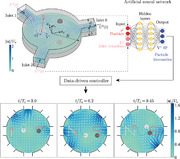

## 1 Introduction

Manipulation of small particles and biological samples plays an important role in different engineering applications and fundamental science. The development of computerized control schemes have facilitated the emerging automated strategies of particle manipulation with limited human interventions. Often, such a manipulation requires a reduced‐order physical model to describe the motion of particles driven by a variety of external fields, for example, magnetic,^[^ ^1^ ^]^ electric,^[^ ^2^ ^]^ acoustic,^[^ ^3^ ^]^ and optical^[^ ^4^ ^]^ ones. However, such models can only be obtained in highly idealized setups that allow multiple assumptions such as point particles, negligible inter‐particles, or particle‐device interactions, and simple geometric settings, etc.^[^ ^5^ ^]^ Lacking such models for general scenarios has considerably limited the application scope of automated particle manipulation. Here, we address this limitation using a data‐driven approach and demonstrate its success in automating a specific technique that exploits hydrodynamics to manipulate particles.

Compared to the external‐field‐driven techniques, hydrodynamic particle manipulation does not need extra power sources but gentle viscous fluid forces on the particles to control their motion. Hence, this technique as exemplified G. I. Taylor's pioneering four‐roll mill device^[^ ^6^ ^]^ does not rely on the specific material or physical properties of particles as often necessitated by field‐based methods. Inspired by the automated four‐roll mill,^[^ ^7^ ^]^ Schroeder and coworkers achieved versatile hydrodynamic particle manipulation in a microfluidic chamber termed Stokes trap^[^ ^5^, ^8^, ^9^ ^]^ using a model predictive control scheme.

Like other field‐driven techniques, automated hydrodynamic manipulation^[^ ^7^, ^9^, ^10^, ^11^, ^12^ ^]^ necessitates a model to characterize the flow‐driven motion of particles, namely, to describe their translational and/or rotational velocities as functions of their states and the control signals, for example, the cylinders' rotational rates of the four‐roll mill. A general model can be obtained by solving the 3D Navier–Stokes or Stokes equations, but is too costly for feasible real‐time control. Reduced‐order models using the Hele–Shaw approximation allowed the manipulations in idealized settings,^[^ ^9^, ^10^, ^13^ ^]^ which however crucially depend on stringent assumptions—a creeping Newtonian flow in a laterally unbounded domain between two closely‐gapped plates together with tracer particles. By choosing the Stokes trap as a model flow device, we present a data‐driven strategy for automated particle manipulation in a general flow configuration relaxing these assumptions. Here, we specifically focus on large particles compared to the device thus featuring considerable hydrodynamic interactions that cannot be conveniently captured by reduced‐order modeling. Using the recorded motion of particles and fluidic control signals as the data, we train an artificial neural network (ANN) that learns the flow physics of hydrodynamically interacting particles in strong confinement to further predict their kinematics subject to varying control signals. We then integrate the ANN into a model predictive path integral (MPPI) controller designed for reinforcement learning tasks.^[^ ^14^, ^15^, ^16^ ^]^ Applying this ANN‐MPPI control strategy in a numerically emulated Stokes trap, we successfully deliver various tasks of hydrodynamic particle manipulation such as targeted assembly, multiplexed path planning and navigating particles through obstacles.

## 2 Virtual Stokes Trap

**Figure 1:**
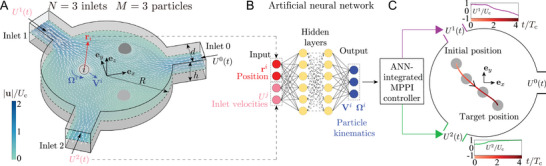
Data‐driven intelligent manipulation of multiple particles in a microfluidic flow at a vanishing Reynolds number Re ≪ 1. A) A 3D disk‐shaped chamber of radius *R* and height *h*, laterally connecting *N* inlet channels of width *d* equally spaced in angle. The flow pattern inside it can be tuned in time for multiplexed motion control of *M* particles of the same radius *a* = 0.1*R* by adjusting the inlets' mean velocities $U^{j}$(*t*) ∈ [− *U*_c_, *U*_c_] with *j* = 1, 2, …, *N*. In this particular *N* = 3 chamber hosting *M* = 3 particles, a snapshot of the flow field in the mid‐plane (*z* = 0) is shown. B) Upon recording the kinematics and positions of particles for sufficiently time, an ANN is trained to predict their translational **V** ^*i*^ and rotational velocities $\Omega^{i}$ (*i* = 1, 2, …, *M*) as functions of their positions **r** ^*i*^ and the inlet velocities $U^{j}$. C) An MPPI controller integrating the ANN model determines the optimal inlet velocities to achieve multiplexed motion control of particles, for example, targeted delivery showcased here of one particle only to avoid visual complexity.

We test and demonstrate by employing the ANN‐MPPI controller for hydrodynamic particle manipulation in a virtual Stokes trap. The emulation is realized by a particle‐resolved flow solver based on Lattice Boltzmann method solving the fluid flow and immersed boundary method capturing the motions of finite‐sized particles (see Appendix). Because Reynolds number Re ≪ 1 in a typical Stokes trap,^[^ ^9^, ^10^ ^]^ the current work considers a creeping flow. We manipulate the motion of *M* spherical particles of the same radius *a* = 0.1*R* inside a disk‐shaped chamber of radius *R* and height *h* = 0.4*R* filled with Newtonian fluids (see **Figure** **1**A), as motivated by the experiments.^[^ ^9^, ^17^ ^]^ Its perimeter is connected to *N* ⩾ 3 side inlets of width *d* = 0.4*R* equally distributed in angle. The mean velocities of fluid flow into these inlets are denoted by $U^{j}$ with 0 ⩽ *j* ⩽ *N* − 1 for the *j*th inlet, satisfying $\sum_{j=0}^{N-1}U^{j}=0$ due to mass conservation. The velocities of the *N* inlets except for the zeroth constitute the Stokes trap's *N* − 1 control variables within the range [ − *U*_c_, *U*_c_]. Controlling the inlet velocities allows for adjusting the flow pattern in the chamber and the motion of particles therein. For demonstration purpose, we constrain the particles to the *z* = 0 mid‐plane, associated with no particle motion in the *z*‐direction due to the top–down symmetry.

## 3 Data‐Driven Feedback Controller

The planar coordinates of the *i*th (*i* = 1, 2, …, *M*) particle are denoted by $r^{i}=\left[r_{x}^{i},r_{y}^{i}\right]$, and its translational and rotational velocities are $V^{i}=\left[V_{x}^{i},V_{y}^{i}\right]$ and Ω_*i*_, respectively. In a creeping flow as considered here, the collection of the particles' translational $\hat{V}=\left[V^{1},\text{…},V^{M}\right]$ velocities depend on their instantaneous positions $\hat{r}=\left[r^{1},\text{…}r^{M}\right]$ and the inlet velocities $\hat{U}=\left[U^{1},\text{…},U^{N-1}\right]$, namely, $\hat{V}=\hat{V}\left(\hat{r},\hat{U}\right)$ and so as the rotational velocities $\hat{\Omega }=\left[\Omega^{1},\text{…},\Omega^{M}\right]$. For a particular Stokes trap, we first numerically calculate $\left[\hat{V},\hat{\Omega }\right]$ as a function of randomly seeded combinations $\left[\hat{r},\hat{U}\right]$. Using the collected data, we train a standard feedforward ANN model typically including 1–3 hidden layers that can calculate the approximate velocities as a function of $\left[\hat{r},\hat{U}\right]$ efficiently (Figure 1B). This approximator will serve as the model for the MPPI controller.

We then briefly describe the MPPI controller for our hydrodynamic manipulation (see Figure 1C). MPPI is a stochastic model predictive control scheme for model‐based reinforcement learning tasks.^[^ ^14^, ^15^ ^]^ At a specific time *t*, MPPI seeks the optimal sequence of actions—inlet velocities here, ${\hat{U}}^{*}=\left({\hat{U}}_{0}^{*},\text{…},{\hat{U}}_{T-1}^{*}\right)$, over *T* steps with each having a time interval of Δ*t*. First, an initial sequence ${\hat{U}}^{*0}$ that ideally approximates ${\hat{U}}^{*}$ is guessed. Second, a number of *K* sequences are sampled around ${\hat{U}}^{*0}$, where the *k*th sequence deviates from ${\hat{U}}^{*0}$ by a small perturbation ${\hat{U}}^{k}-{\hat{U}}^{*0}=\varepsilon^{k}=\left(\varepsilon_{0}^{k},\text{…},\varepsilon_{T-1}^{k}\right)$ representing an exploration noise. The components of ***ε*** follows the normal distribution, namely, $\varepsilon_{l}∼N\left(0,\sigma^{2}I\right)$ with *l* = 0, …, *T* − 1, where σ^2^ denotes the exploration variance and I the identity matrix of size *N* − 1. Accordingly, the optimal sequence ${\hat{U}}^{*}$ will be identified as a weighted sum of all sampled sequences ${\hat{U}}^{*}={\hat{U}}^{*0}+\sum_{k=1}^{K}w^{k}\varepsilon^{k}$ subject to $\sum_{k=1}^{K}w^{k}=1$, where the weight $w^{k}$ quantifi=es the relative importance of the *k*th sequence. To determine $w^{k}$, we first use the ANN model to evolve the sequence in the time window [*t*, *t* + *T*Δ*t*] and calculate a cost value *S* ^*k*^ to quantify how far the evolved trajectory deviates from the objective. We then obtain

(1) $$\begin{array}{cc} W^{k} & =\exp\left[-S^{k}-\frac{\sigma^{-2}}{2}\sum_{l=0}^{T-1}{\hat{U}}_{l}^{*0}\cdot \left({\hat{U}}_{l}^{*0}+2\varepsilon_{l}^{k}\right)\right] \end{array}$$

leading to $w^{k}=W^{k}/\sum_{k=1}^{K}W^{k}$. In particular, the cost $S^{k}=\sum_{l=1}^{T}\psi \left[\hat{r}\left(t\right),{\hat{r}}^{k}\left(t^{\prime }=t+l\Delta t\right)\right]$ sums the instantaneous cost functions ψ within the sampled time window *t*′ ∈ [*t*, *t* + *T*Δ*t*]. The form of ψ(*t*′) shall be designed case by case for different manipulative tasks. In the current work for path‐planning and navigation, ψ involves only the positions of particles.

## 4 Results

### 4.1 Steering Particles to Follow Individual Trajectories

**Figure 2:**
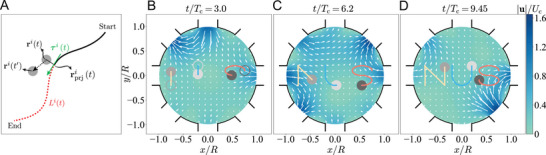
Guiding particles such that each executes its respectively assigned target trajectory. Here, we steer three particles to trace the letters “N,” “U,” and “S” and finalize their journeys simultaneously. A) The solid and dashed curves indicate the completed and unfinished parts of a prescribed trajectory for the *i*th particle at time *t*, when the contour length of the latter is *L* ^*i*^(*t*). The two parts are separated by the projection $r_{\mathrm{prj}}^{i}\left(t\right)$ of the particle's position **r** ^*i*^(*t*) onto the trajectory. B–D) Instantaneous positions and past trajectories of the particles at *t*/*T*_c_ = 3.0, 6.2, and 9.45. The hollows circles in (B) mark the initial positions of particles. The vectors and contour color indicate the mid‐plane velocity fields $u/U_{c}$ at *z* = 0.

First, we steer *M* particles to follow an individual target trajectory *f* ^*i*^(**r**) = 0 (*i* = 1, …, *M*) simultaneously. We use a two‐component instantaneous cost function ψ consisting of

(2) $$\psi_{1}=\sum_{i=1}^{M}\left|f^{i}\left(r^{i}\left(t^{\prime }\right)\right)\right|,\psi_{2}=-\frac{\delta \hat{r}\cdot \hat{L}\left(t\right)}{∥\delta \hat{r}∥∥\hat{L}\left(t\right)∥}$$

where $\delta \hat{r}=\left[\delta r^{1},\text{…},\delta r^{M}\right]$ with $\delta r^{i}=\left[r^{i}\left(t^{\prime }\right)-r^{i}\left(t\right)\right]\cdot \tau^{i}\left(t\right)$ representing the projected travelling distance of the *i*th particle along its desired trajectory within the time window [*t*, *t*′] as shown in **Figure** **2**A. Here, $\tau^{i}\left(t\right)$ is the tangent vector at the projected position of the *i*th particle's position **r** ^*i*^(*t*) onto its desired trajectory. $\hat{L}\left(t\right)=\left[L^{1},\text{…},L^{M}\right]\left(t\right)$ with *L* ^*i*^(*t*) is the contour length of the remaining (uncompleted) part of the desired trajectory at time *t*, where the completed and remaining parts are separated by the projection $r_{\mathrm{prj}}^{i}\left(t\right)$. The first cost function ψ_1_ guides the particles to follow their respective target trajectories, and the second function ψ_2_ guarantees all particles to complete their journeys at the same time.

Using a Stokes trap of *M* = 8 inlets, Figure 2 shows that the MPPI controller based on the cost function Equation (2) enables the steering of three particles to successfully trace out the letters “NUS” simultaneously (see Video S1, Supporting Information), that is, the particles start and finish the tracing of individual letters simultaneously. This example showcases the capability of this ANN‐MPPI approach in the precise control of multiple particles both spatially and temporally, empowering complex and simultaneous manipulations of particles in subsequent applications.

### 4.2 Targeted Assembly of Particles

Having guided the particles to follow individual trajectories, we then attempt targeted assembly of particles as a powerful mean to synthesize colloidal molecules and superstructures.^[^ ^18^, ^19^ ^]^ For a numerical demonstration, we address how to gather particles together without modeling the inter‐particle bondage, considering that protein‐coated particles brought sufficiently close can form a stable assembly.^[^ ^9^, ^20^ ^]^

**Figure 3:**
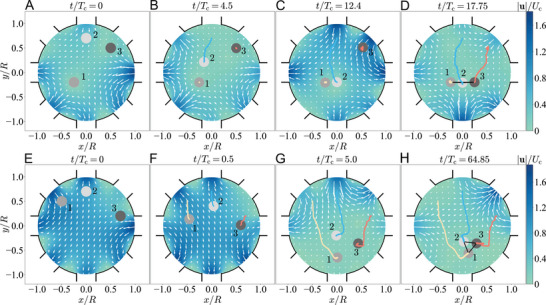
Targeted assembly of three particles into a line array (top row) or an equilateral triangle cluster (bottom row) featured by a uniform surface‐to‐surface distance of 0.5*a*. This relatively large distance is adopted here to ease the numerical demonstration. A) Initial positions of particles. B,C) Controlling particles 1 and 3 to stay still but particle 2 next to particle 1. D) Freezing particles 1 and 2 while moving particle 3 to assemble them into a line array. E–H) Moving three particles simultaneously to form an equilateral triangle cluster.

In **Figure** **3**A–D, we demonstrate the sequential assembly of three particles numbered 1–3 (see Video S2, Supporting Information) using the cost functions described in Appendix. First, the inlet flows are controlled such that particle 1 is held fixed in space throughout the assembly process, similar to the use of an optical tweezer or a micro‐pipette aspiration in trapping or confining a particle or biological cell. Particle 2 is then transported next to particle 1 (Figure 3B,C), a representative operation conducted to initiate particle–particle interactions in scenarios such as drop coalescence,^[^ ^21^ ^]^ vesicle fusion,^[^ ^22^ ^]^ and cellular communications.^[^ ^23^ ^]^ Subsequently, steering particle 3 to approach particle 2 while freezing particles 1–2 allows the three to form a line assembly (Figure 3D). This example showcases the current approach as a versatile tool for building assembly of particles encoding a specific ordering, for instance, the surfactant‐like or barcode‐mimicking colloidal chain.^[^ ^24^ ^]^

Besides the sequential chain formation, the particles can also be assembled into structures of different shapes simultaneously, namely, all particles are brought into close contact at the same time. We illustrate in Figure 3E–H the simultaneous assembly of particles in the shape of an equilateral triangle (see Video S3, Supporting Information) based on the cost functions given in the Appendix. As a remark, the manipulation via fluid flows alone here is contactless and noninvasive, without relying on optical, magnetic, or other physical properties of the particles typically required by other particle manipulation techniques.

### 4.3 Path Planning of a Particle Assembly

**Figure 4:**
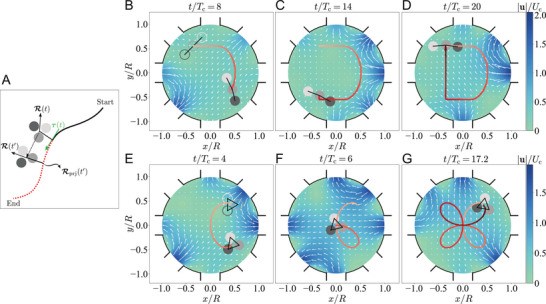
Path planning of a three‐particle assembly. A) Schematic similar to Figure 3A but focusing on the centroid position of the cluster. B–D) Guiding a line assembly to follow letter “D.” E–G) Guiding a triangular assembly to follow a clover‐shaped path. The hollow circles in (B) and (E) denote the initial positions of particles.

The ANN‐MPPI‐controlled Stokes trap cannot not only produce different particle assemblies but also control the motion of the assemblies. We illustrate this capability by controlling a three‐particle line assembly to trace out the letter “D” in **Figure** **4**B–D (see Video S4, Supporting Information). As a second example, in Figure 4E–G the particle assembly shaped in equilateral triangle nicely executes a convoluted, clover‐shaped path (see Video S5, Supporting Information). Note that the particles here are not bonded but free to move apart. Hence, it is remarkable that the forces responsible for both holding the particles together as an assembly and steering itself during the whole course are solely generated by the fluid flows modulated by the ANN‐MPPI controller.

To achieve the above tasks, we use a three‐component instantaneous cost function ψ = ω_1_ψ_1_ + ω_2_ψ_2_ + ω_3_ψ_3_ as

(3a) $$\begin{array}{cc} \psi_{1} & =∥R\left(t^{\prime }\right)-R_{\mathrm{prj}}\left(t^{\prime }\right)∥/R \end{array}$$

(3b) $$\begin{array}{cc} \psi_{2} & =-\mathrm{sgn}\left(\left[R\left(t^{\prime }\right)-R\left(t\right)\right]\cdot \tau \left(t\right)\right) \end{array}$$

(3c) $$\begin{array}{cc} \psi_{3} & =\sum_{i,j}^{i\neq j}|∥r^{i}\left(t^{\prime }\right)-r^{j}\left(t^{\prime }\right)∥-d_{\mathrm{tar}}^{ij}|/R \end{array}$$

where R denotes the centroid of assembly, $R_{\mathrm{prj}}$ indicates the projection of R onto the prescribed path, and ***τ*** the latter's corresponding tangent vector at $R_{\mathrm{prj}}$ (see Figure 4). We design the cost functions ψ_1_ and ψ_2_ for guiding the centroid to move along the desired path, where the former reduces the centroid‐path distance and the latter allows the centroid to follow the path profile. Besides, ψ_3_ is added to preserve the inter‐particle distances and hence the shape of the particle assembly. Here, the weights ω_1_ = 3000, ω_2_ = 30 and ω_3_ = 5000 are used.

### 4.4 Guiding Particles through Obstacles

**Figure 5:**
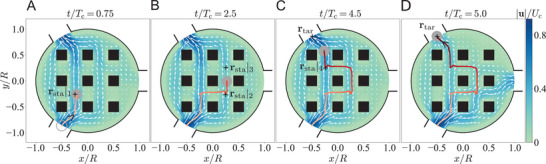
Navigating one particle in a three‐inlet chamber including a uniform grid of square pillars each of size 0.2*R*. The center‐to‐center distance between every two neighboring pillars is 0.5*R*. The particle is directed to enter (resp. leave) the chamber via the southwest (resp. northwest) inlet. **r**_sta_|_*l*_ denotes the intermediate stations introduced to guide the particle.

Microfluidic chambers with obstacles have been adopted as a controlled testbed to examine transport phenomena of particulate media in porous media.^[^ ^25^, ^26^ ^]^ In particular, porous structures typically complicate the motion of particles and hence make it difficult to steer them. Here, we explore using intelligent hydrodynamic manipulation for collision‐free navigation of particles in a “porous” environment depicted in **Figure** **5**. This setting is featured by a 3 × 3 uniform lattice of square pillars each of width 0.2*R*, with a center‐to‐center distance of 0.5*R* between every two neighboring pillars. Unlike the above‐shown multiplexed manipulations, here, we navigate a single particle to focus on collision avoidance. Despite the complex particle‐pillar hydrodynamic interaction as captured by the ANN, the degrees of freedom of the particle motion remain two, thus allowing an *N* = 3 chamber for motion control as shown in Figure 5. To facilitate the navigation, we set up $\tilde{N}=5$ intermediate stations **r**_sta_|_*l*_ ($l=1,\text{…},\tilde{N}$) guiding the particle to follow a polyline toward the target $r_{\mathrm{tar}}\equiv r_{\mathrm{sta}}{|}_{\tilde{N}}$. Hence, the particle enters the southwestern inlet of the chamber and leaves it from the northwestern one via these targets consecutively (see Video S6, Supporting Information). The cost function for targeting the *l*th station reads

(4) $$\begin{array}{cc} \psi (l) & =\omega \frac{∥r_{\mathrm{sta}}{{|}_{l}-r^{1}\left(t^{\prime }\right)∥}^{2}}{∥r_{\mathrm{sta}}{{|}_{l}-r^{1}\left(t\right)∥}^{2}} \end{array}$$

with ω = 1000. Note that we have not introduced a cost function to penalize and thus avoid the particle collision with the obstacles. Instead, the MPPI controller abandons any collision‐leading sampling sequences by setting their corresponding weights $W^{k}$ (Equation (1)) to zero.

## 5 Conclusion and Discussion

Automated hydrodynamic control has been an emerging technique for versatile manipulation of micro‐scale particulate media. Previous studies utilizing feedback control necessitate a reduced‐order model to describe the flow‐driven particle motion. The model, though indispensable, is however not accessible for general microfluidics likely featuring finite inertia,^[^ ^27^ ^]^ non‐Newtonian fluids,^[^ ^28^ ^]^ non‐spherical particles and cells,^[^ ^29^ ^]^ strong hydrodynamic interactions,^[^ ^30^ ^]^ or complex channels. In this work, we replace the generally inaccessible model with a readily trainable ANN with sufficient data. Then, we integrate it to an MPPI controller to achieve a plethora of targeted and directed control of particles in a numerically‐emulated microfluidic device, demonstrating both spatial and temporal controllability of high precision.

**Figure 6:**
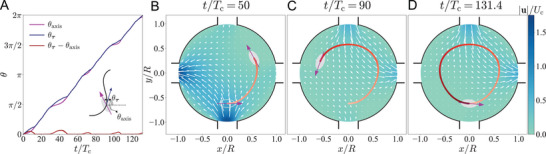
Simultaneously controlling the motion and orientation of a prolate particle of aspect ratio 2: its centroid executes a perturbed circular trajectory while its major axis is consistently aligned with the trajectory. $\theta_{\tau }$ is the angle between the particle's translational velocity and the $e_{x}$ axis, while θ_axis_ denotes that between the particle's major axis and the *x*‐axis.

To demonstrate using the learned hydrodynamics of interacting particles for their versatile motion control, our current setting has been limited to a Newtonian creeping flow. Upon relaxing the limitation, we expect the control strategy to excel also in other general scenarios. **Figure** **6**, for example, showcases manipulating a prolate to follow a perturbed circular path with the former's major axis persistently aligned with the path. Throughout this work, we have exploited the instantaneity of Stokes flow to use the simplest feedforward ANN without considering how particle kinematics depends on the past information (e.g., the particle positions or inlet velocities). However, this dependence would arise in inertial^[^ ^27^ ^]^ or viscoelastic^[^ ^28^ ^]^ microfluidic flows or other scenarios involving deformable structures such as filaments,^[^ ^31^, ^32^ ^]^ cells,^[^ ^33^ ^]^ or fluid interfaces.^[^ ^34^ ^]^ This consequently calls for using ANNs with memory, for example, recurrent neural networks^[^ ^35^ ^]^ to characterize particle kinematics.

The numerically demonstrated data‐driven intelligent Stokes trap can be readily realized in experiments considering the already mature utilization of Stokes traps^[^ ^5^, ^8^, ^9^, ^10^, ^11^, ^12^ ^]^ and that ANNs handle numerical and physical data indistinguishably. We also anticipate that the data‐driven approach may obviate certain equipment calibrations. The original setting requires calibrating the relation between the flow rate in a tubing and the applied voltage of a pressure regulator, because the former is varied by adjusting the latter that determines the pressure. Such calibrations are unnecessary because ANNs directly map the applied voltage onto the particles' kinematics. Another related benefit of using ANN lies in its ability to model, as a “black box,” systematic imperfection and malfunction of certain device parts, which would enable the ANN‐based control without actually fixing the equipment glitches.

Future directions for automated hydrodynamic manipulation include applying reinforcement learning to learn the dynamics while controlling as demonstrated recently,^[^ ^36^ ^]^ incorporating the principle of symmetry‐based manipulation,^[^ ^37^ ^]^ manipulating motile microorganisms or synthetic microswimmers in spirit of exploring or endowing artificial intelligence in these swimmers,^[^ ^38^, ^39^, ^40^, ^41^, ^42^, ^43^, ^44^ ^]^ and integrating deep‐learning‐enabled image classification^[^ ^45^ ^]^ for automated cell sorting. We also envision that machine learning for particle manipulation is not limited to hydrodynamic control but can be conveniently adopted for other external‐field‐directed techniques. The extrapolation has been exemplified by the recent studies employing reinforcement learning to manipulate particles using an optical^[^ ^46^ ^]^ or acoustic field.^[^ ^47^, ^48^ ^]^ Taken together, we anticipate a surge of using machine learning to endow microfluidics with intelligence.^[^ ^49^, ^50^, ^51^ ^]^

## Conflict Of Interest

The authors declare no conflict of interest.

## Supporting information

Supplemental Video 1

Supplemental Video 2

Supplemental Video 3

Supplemental Video 4

Supplemental Video 5

Supplemental Video 6

## Data Availability

The data that support the findings of this study are available from the corresponding author upon reasonable request.

## A.1 Numerical Methods

We build the virtual Stokes trap using a 3 flow solver based on Lattice Boltzmann method (LBM) and immersed boundary method (IBM): the former is adopted to solve the flow and latter to capture the interfaces of moving particles. Besides, no‐slip boundary conditions on the chamber walls and square pillars are imposed using the standard bounce‐back scheme of LBM. The LBM–IBM solver enables explicitly resolving the fluid motion around particles, as well as the flow between particles and chamber walls. Hence, inter‐particle and particle‐wall hydrodynamic interactions are captured directly as a part of solution. Details of the LBM–IBM implementation are referred to ref. [52]. Note that since we use the solver to emulate a virtual microfluidic device instead of examining specific flow physics, we do not pursue an especially high numerical resolution in space: the diameter 2*a* of particle spans 12 LBM grids of size d*x* in most simulations, and 24 grids for the case with obstacles (Figure 5). Besides, we have imposed a repulsive force^[^ ^53^, ^54^ ^]^ on particles when they are in close proximity to a nearby particle or channel wall. Admittedly, this repulsion is partially ad hoc, though also encoding a physical origin of lubrication force that is typically underestimated due to an insufficient grid resolution.

Using this LBM‐IBM implementation, we do not actually solve the Stokes equation but the incompressible Naiver–Stokes equation with small but finite inertia, that is, 0 < Re = ρ*U*_c_ *R*/μ that is ideally much smaller than unity to approach the considered Stokes flow limit, where ρ and μ denote the density and dynamic viscosity of the fluid, respectively. Here, Re also sets the numerical time step d*t* following d*t*/*T*_c_ = (d*x*/*R*)^2^(τ_LBM_ − 0.5)Re/3 where the dimensionless relaxation time τ_LBM_ = *O*(1).

To approximate the Re = 0 limit, we set Re = 0.1 corresponding to a particle Reynolds number of Re_p_ = ρ*U*_c_ *a*/μ = 0.01 considering the fixed size ratio *a*/*R* = 0.1 throughout the study. In typical flows that vary smoothly in time, such a small Re_p_ is safely below the threshold required to neglect inertia, hence allowing Navier–Stokes solvers to address Stokes flows as commonly used.^[^ ^55^ ^]^ However, this strategy needs to be modified for a flow featuring significant time variation as shown here. In our case, the inlet velocities $\hat{U}/U_{c}$ are changed as the control signal at a constant time interval Δ*t* (that determines how often the controller changes the inlet velocities). A sudden flow variation upon every adjustment will boost the acceleration term Re∂u∼/∂t∼ that would otherwise be zero in an exact Stokes flow. To eliminate this artificially large acceleration, we seek a stationary state every time a new control signal $\hat{U}/U_{c}$ is imposed: we hold the positions of particles but update their velocities and the flow field iteratively to be time‐independent. This iterative process is time costly and is used only at the first time step upon the adjustment.

Besides, we choose the control step Δ*t*/*T*_c_ = 0.05 throughout the work as guided by the original implementations of Stokes trap.^[^ ^9^, ^17^ ^]^ In the experiments, the controller modulates at a frequency of 30 Hz, resulting in a dimensionless control step Δ*t*/*T*_c_ ≈ 0.04, where *T*_c_ is calculated according to the experimental values.

## A.2 Sequential Line Assembly

To form the sequential line assembly shown in the top row of Figure 3, we deliver particles to the target positions corresponding to the assembly. We use a two‐stage cost function ψ: the first stage for steering particle 2 to approach particle 1 that is held still simultaneously (Figure 3B,C), and the second stage for attaching particle 3 to the assembly of particles 1 and 2 (Figure 3D). The first‐stage cost function ψ = ω_1_ψ_1_ + ω_2_ψ_2_ + ω_3_ψ_3_ involves

(A1a)
$$\begin{array}{cc} \psi_{1} & =\frac{∥r_{\mathrm{tar}}^{2}-r^{2}\left(t^{\prime }\right){∥}^{2}}{∥r_{\mathrm{tar}}^{2}-r^{2}{\left(t\right)∥}^{2}} \end{array}$$

(A1b)
$$\begin{array}{cc} \psi_{2} & =∥r_{\mathrm{tar}}^{1}-r^{1}\left(t^{\prime }\right){∥}^{2}/R^{2} \end{array}$$

(A1c)
$$\begin{array}{cc} \psi_{3} & =∥r_{\mathrm{ini}}^{3}-r^{3}\left(t^{\prime }\right){∥}^{2}/R^{2} \end{array}$$

where $r_{\mathrm{ini}}^{i}$ and $r_{\mathrm{tar}}^{i}$ denote the initial and target positions of the *i*th particle, respectively. Note $r_{\mathrm{ini}}^{1}=r_{\mathrm{tar}}^{1}$. Here, ψ_2_ and ψ_3_ enforce the stationarity of particles 1 and 3, respectively. The weights ω_1_ = 100, ω_2_ = 10^5^ and ω_3_ = 2 × 10^4^. The second‐stage cost function can be designed similarly.

## A.3 Build a Triangular Assembly Simultaneously

Similar to the line assembly, we first specify the target positions ${\hat{r}}_{\mathrm{tar}}=\left[r_{\mathrm{tar}}^{1},r_{\mathrm{tar}}^{2},r_{\mathrm{tar}}^{3}\right]$ of particles forming a triangular cluster. We design the cost function ψ = ω_1_ψ_1_ + ω_2_ψ_2_ comprising

(A2)
$$\psi_{1}=\frac{∥{\hat{r}}_{\mathrm{tar}}-\hat{r}\left(t^{\prime }\right){∥}^{2}}{∥\Delta \hat{r}{∥}^{2}},\psi_{2}=-\frac{\left[\hat{r}\left(t^{\prime }\right)-\hat{r}\left(t\right)\right]\cdot \Delta \hat{r}}{∥\hat{r}\left(t^{\prime }\right)-\hat{r}\left(t\right)∥∥\Delta \hat{r}∥}$$

where $\Delta \hat{r}={\hat{r}}_{\mathrm{tar}}-\hat{r}\left(t\right)$. The second component, ψ_2_ ∈ [ − 1, 1], drives the particles toward their goals as straightforward as possible; the optimal scenario of ψ_2_ = −1 occurs when every particle lies on the path connecting its instantaneous (at *t*) and target position. Here, *w*_1_ = 500 and *w*_2_ = 30 are used.
