# Corrigendum: Data‐Driven Intelligent Manipulation of Particles in Microfluidics

**DOI:** 10.1002/advs.202309675

**Published:** 2024-03-06

**Authors:** Wen‐Zhen Fang, Tongzhao Xiong, On Shun Pak, Lailai Zhu

**Affiliations:** ^1^ Department of Mechanical Engineering National University of Singapore Singapore 117575 Singapore; ^2^ Department of Mechanical Engineering Santa Clara University Santa Clara CA 95053 USA; ^3^ Present address: Key Laboratory of Thermo‐Fluid Science and Engineering, MOE Xi'an Jiaotong University Xi'an 710049 China

## Corrigendum

This corrigendum provides a correction to the acknowledgement. Its correct version is given below.

